# Changes in Socioeconomic Status as Predictors of Cardiovascular Disease Incidence and Mortality: A 10-Year Follow-Up of a Polish-Population-Based HAPIEE Cohort

**DOI:** 10.3390/ijerph192215411

**Published:** 2022-11-21

**Authors:** Magdalena Kozela, Maciej Polak, Urszula Stepaniak, Martin Bobak, Andrzej Pająk

**Affiliations:** 1 Department of Epidemiology and Population Studies, Institute of Public Health, Jagiellonian University Medical College, 31-008 Krakow, Poland; 2 Department of Epidemiology and Public Health, University College London, London WC1E 6BT, UK

**Keywords:** socioeconomic status, social mobility, cardiovascular disease, mortality, incidence, Central and Eastern Europe

## Abstract

Although the inverse association between socioeconomic status (SES) and cardiovascular disease (CVD) is well established, research on the effect of changes in the SES throughout life on CVD risk in populations with different social backgrounds remains scarce. This study aimed to assess the relationship between childhood SES, adulthood SES, and changes in SES over time, and CVD incidence and mortality in a Polish urban population. In addition, the predictive performance of the SES index was compared with education alone. A cohort study with a 10-year follow-up was conducted, in which a random sample of 10,728 residents in Kraków aged 45–69 years were examined. The SES was assessed at baseline using data on education, parents’ education, housing standard at the age of 10 years, professional activity, household amenities, and difficulties in paying bills and buying food. SES categories (low, middle, and high) were extracted using cluster analyses. Information on new CVD cases was obtained from questionnaires in subsequent phases of the study and confirmed by reviewing clinical records. Data on deaths and causes were obtained from the residents’ registry, Central Statistical Office, and the participants’ families. The effect of the SES index on the risk of CVD was assessed using Cox proportional hazard models. In male and female participants, the CVD incidence and mortality were observed to be 27,703 and 32,956 person-years (384 and 175 new CVD cases) and 36,219 and 40,048 person-years (159 and 92 CVD deaths), respectively. Childhood SES was not associated with CVD incidence and mortality. A protective effect of high adulthood SES against CVD mortality was observed in men and women (HR = 0.59, 95% CI = 0.31–0.97; HR = 0.33, 95% CI = 0.14–0.75, respectively). In women, downward social mobility was related to 2.24 and 3.75 times higher CVD incidence and mortality, respectively. In men, a protective effect against mortality was observed in upward mobility (HR = 0.50, 95% CI = 0.29–0.84). Model discrimination was similar for the SES index and education alone for the association with CVD incidence. In women, the SES index was a slightly better predictor of CVD mortality than education alone (C-index = 0.759, SE = 0.0282 vs. C-index = 0.783, SE = 0.0272; *p* = 0.041). In conclusion, high adulthood SES, but not childhood SES, may be considered to be a protective factor against CVD in urban populations in high-CVD-risk regions. No effects of critical periods in early life were observed on CVD risk. In later life, social mobility was found to affect CVD mortality in both men and women. In men, a protective effect of upward mobility was confirmed, whereas in women, an increased CVD risk was related to downward mobility. It can be concluded that CVD prevention may be beneficial if socioeconomic potentials are strengthened in later life.

## 1. Introduction

The association between socioeconomic status (SES) and cardiovascular disease (CVD) is well known [1,2]. Knowledge of this association has been accumulating for several years, and recent data from stable, high-income countries confirm that socioeconomic inequalities in CVD mortality remain a major public health problem [3]. Furthermore, the gap in the evidence limits the use of this knowledge in the prevention of CVD [4,5]. The latest European Society of Cardiology Guidelines on CVD prevention in clinical practice indicate that more evidence from different risk regions is needed to consider the inclusion of socioeconomic factors in risk prediction models [6]. The findings of research in Central and Eastern Europe remain inconclusive. On the one hand, extending the recommended risk prediction algorithm, known as Systematic Coronary Risk Evaluation (SCORE), to include education and marital status failed to improve its prognostic performance substantially in population-based cohorts. On the other hand, the inclusion of seven quick, noninvasive, and cheap predictors, including socioeconomic characteristics, improved the prediction accuracy of the SCORE model [7,8]. The results of the MORGAM study showed that the inclusion of education in the CVD risk prediction model might be beneficial in men, especially in Nordic and Eastern European countries, but not in women [9].

This ambiguity in the significance of SES characteristics in CVD risk assessment may be due to differences in SES assessment methods. For example, education is a widely used indicator of SES in population studies and is known to be related with CVD mortality; however, its use is undoubtedly a simplification of the assessment. Education alone, as well as other simplified single measures of SES, may not necessarily reflect multidimensional SES characteristics. In studies on social inequalities in health, there is still no consensus on the best single measure of SES [10,11]. Given the continuous social development and more universal access to higher education, education may not differentiate population sufficiently, and SES assessments need to be more sophisticated to better reflect the complexity of the construct.

Furthermore, the SES affects the lives and health of individuals from early childhood throughout their life. Socioeconomic circumstances increase CVD risk through various mechanisms. On the one hand, childhood might be the critical period in which low socioeconomic conditions determine later poor health; on the other hand, accumulating lifelong exposure to low SES may be even more important [11]. The effect of changes in SES throughout life on CVD risk remains unclear.

In this context, Central and Eastern Europe provide an interesting setting for studying the SES. In the 1990s and early 2000s, Central and Eastern European countries experienced political and economic transitions. Hence, many people experienced changes in social position. During the same time, after a long period of high and increasing CVD mortality, the trend was reversed.

This study aimed to determine the relationship between childhood SES, adulthood SES, and changes in SES over time (social mobility), and CVD incidence and mortality in high-cardiovascular-risk region, with particular emphasis on the comprehensive assessment of the SES. In addition, the predictive performance of the SES index was compared with education alone.

## 2. Materials and Methods

### 2.1. Data

The Polish cohort of the Health, Alcohol and Psychosocial factors In Eastern Europe (HAPIEE) study was established in Krakow. A random population sample of men and women aged 45–69 years at baseline, stratified by gender and 5-year age groups, was contacted. The baseline examination was completed between 2002 and 2005. In total, 10,728 participants were recruited (with a 61% response rate), and all participants gave written consent. At baseline, trained nurses interviewed the participants in their homes using a standardized questionnaire. Detailed information on socioeconomic characteristics, health, and health behaviors was collected. Then, all participants were invited to the clinic for anthropometric and blood pressure measurements and blood sample collection for biochemical tests [12].

### 2.2. SES

Childhood SES was assessed using questions about the level of education of the participants’ parents and housing standard at about 10 years of age (cold tap water, hot tap water, radio, fridge, own kitchen, and own toilet). Adulthood SES was assessed using the information on the participants’ education, professional activity, household amenities, and difficulties in paying bills and buying food. The SES index was constructed as described in detail elsewhere [13]; a summary is provided below.

The participants were classified into homogeneous groups of childhood SES and adulthood SES using a two-stage clustering algorithm. Three groups of childhood SES (low, middle, and high) were generated, with reasonable evidence of cluster structure (silhouette coefficient (s(i)) = 0.51). Similarly, three groups of adulthood SES (low, middle, and high) were identified (s(i) = 0.55). Using the data on childhood and adulthood SES of each participant, the following social mobility categories were generated: (1) always low; (2) downward mobility if childhood SES was middle or high but adulthood SES was low; (3) upward mobility if childhood SES was low and adulthood SES was middle or high; and (4) always middle or high. The description of the SES clusters and the distribution of the characteristics used for the SES index are presented in Supplementary Table S1.

### 2.3. Incidence and Mortality Assessment

New cases of nonfatal CVD events, i.e., myocardial infarction, stroke, coronary artery bypass graft, percutaneous coronary interventions, and unstable coronary disease (confirmed using coronary angiography), were identified in reexamination interviews (2006–2008) and in three postal questionnaires (2005–2006, 2008–2010, and 2012–2013). Both reexamination interviews and postal questionnaires had an identical series of questions about whether the participant had had myocardial infarction, stroke, coronary angiography, coronary artery bypass graft, or percutaneous coronary interventions since the last contact. In each case, when the participant reported the presence of the disease, the study team verified the clinical diagnosis by reviewing the medical documentation. Data on deaths by cause were obtained from the mortality register of the residents of the city of Krakow, Central Statistical Office, and by contacting the participants’ families. CVD deaths were accepted for codes from I.00 to I.99, according to the 10th revision of International Statistical Classification of Diseases and Health Problems (ICD-10). For each participant, their status at the end of the observation was determined, and the exact survival time was calculated. The follow-up was completed on 31 December 2013. For participants who were lost at follow-up, the censorship date was considered as the date of the last contact.

### 2.4. Covariates

Covariates measured at baseline included age, marital status (married/cohabiting vs. single/separated/divorced/widowed), and five main CVD risk factors according to the latest ESC guidelines on clinical practice: smoking status, obesity (BMI from clinical examination in kg/m^2^ ≥ 30), hypercholesterolemia (low density lipoprotein concentration ≥3 mmol/L or receiving lipid-lowering treatment), hypertension (blood pressure ≥140/90 mm Hg or receiving hypotensive treatment), and diabetes (fasting plasma glucose ≥7 mmol/L or having diabetes diagnosed by the doctor).

### 2.5. Statistical Analysis

Continuous variables were reported as means (standard deviation) or medians (first and third quartiles (Q1–Q3)), as appropriate. The Kolmogorov–Smirnov test was used to test the normal distribution of variables. Categorical variables were reported as numbers and percentages. The differences in the variables were tested using Student’s test or the analysis of variance and the χ^2^ test. The relationships between SES, and CVD incidence and mortality were assessed using multivariable Cox proportional hazard models. Separate models for the SES index in childhood, the SES index in adulthood, social mobility, and education alone were fitted. Two sets of models were conducted: model A—adjusted for age and marital status; and model B—with additional adjustment for main CVD risk factors, i.e., obesity, hypertension, hypercholesterolemia, smoking, and diabetes. The results of Cox regression were reported as hazard ratios (HRs) with 95% confidence intervals (CIs). All analyses were carried out in sex groups. The goodness-of-fit of the preformed model with the SES index and that of the model with education alone were compared using Harrell’s C-index. Two-sided *p*-Values < 0.05 were considered to be statistically significant. Analyses were performed using IBM Corp. software released in 2021 (IBM SPSS Statistics for Windows; version 28.0.; IBM Corp, Armonk, NY, USA) or R Core Team (2013) (R: A language and environment for statistical computing; R Foundation for Statistical Computing, Vienna, Austria).

## 3. Results

The observation for CVD incidence included 27,703 and 32,956 person-years in men and women, respectively. The date of censorship for nonfatal CVD cases was the latest date the participant was confirmed to be free of CVD. A total of 384 and 175 new CVD cases were observed in men and women, respectively. The date of censorship for mortality analyses was 31 December 2013, which included 36,219 and 40,048 person-years in men and women, respectively. A total of 159 and 92 CVD deaths were reported in men and women, respectively (Table 1).

The mean baseline age of participants was 56 years (SD = 6.9). In this study, men were more likely to be married or cohabiting (87.5% vs. 67.5%; *p* < 0.001) than women. The distribution of childhood SES was similar in men and women, with about one-fourth of the participants having a high SES. Sex differences in adulthood SES distribution were more pronounced, as a higher number of men had a high SES. Similarly, the distribution of social mobility was more advantageous in men, who had at least a middle SES significantly more often (50% vs. 45%; *p* < 0.001) throughout their life and experienced upward mobility more frequently (28% vs. 24%; *p* < 0.001). Men were more likely to smoke and have hypertension and diabetes (*p* < 0.001), whereas women were more often obese (32.3% vs. 25.3%; *p* < 0.01). No significant differences were observed in the prevalence of hypercholesterolemia (Table 1). Detailed descriptive statistics for age, marital status, smoking, obesity, hypertension, diabetes, hypercholesterolemia, SES in childhood and adulthood, social mobility, and sex are presented in Supplementary Tables S2 and S3.

The association between childhood SES and CVD incidence was not significant (Table 2). In the case of adulthood SES, compared with participants with low SES, after adjustment for age and marital status, high SES was related to a 32% reduced risk of CVD (*p* < 0.05) in men and 60% (*p* < 0.001) in women. Further adjustment for main CVD risk factors attenuated the association in men, so it was insignificant in fully adjusted models. In women, the attenuation was slighter, and compared with women with low SES, more than 40% reduced risk of CVD was observed in women with high SES (HR of 0.57 and 95% CI of 0.35–0.94). In the assessment of social mobility, although a reduction in CVD risk was observed in men with always middle or high SES and in men who experienced upward social mobility after adjustment for age and marital status, these associations were not confirmed in the fully adjusted models. However, in women, associations with social mobility were observed only on different levels of exposure. A higher than twofold increase in the risk of CVD was observed for downward mobility (HR of 2.24 and 95% CI of 1.25–4.02 in fully adjusted models). In women, no associations between always middleor high SES or upward mobility and CVD incidence were observed (Table 2).

Associations between SES and CVD mortality are presented in Table 3. Similar to CVD incidence, no statistically significant differences between childhood SES and CVD mortality were observed in the fully adjusted models. However, for adulthood SES and social mobility, associations with mortality were consistent and even more pronounced than those with incidence. After adjusting for age and marital status, high adulthood SES was related to a 56% reduced risk of CVD death (*p* < 0.001) in men and 78% (*p* < 0.001) in women. In the fully adjusted models, the HRs were only slightly attenuated (HR of 0.59 and 95% CI of 0.36–0.95; HR of 0.33 and 95% CI of 0.14–0.75 for men and women, respectively). The significant effect of social mobility on CVD mortality was observed in both men and women. In men, a strong protective effect of maintaining middle or high SES and upward mobility was observed (HR of 0.55 and 95% CI = 0.31–0.97 for upward mobility vs. always-low SES). The effect size was similar for maintaining middle or high SES and upward mobility and indicated about 50% risk reduction compared with always-low SES. In women, the relationship between social mobility and CVD mortality was demonstrated only as the negative impact of deteriorating SES (HR of 3.75 and 95% CI = 1.43–9.88 for downward mobility vs. always-low SES).

**Figure 1:**
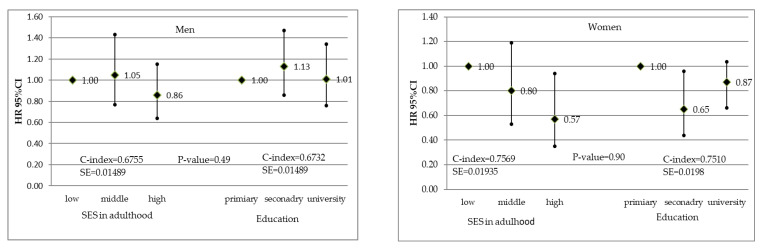
Associations between the socioeconomic status index in adulthood and education, and cardiovascular disease incidence in men and women. Hazard ratios adjusted for age, marital status, obesity, hypertension, hypercholesterolemia, smoking, and diabetes.

**Figure 2:**
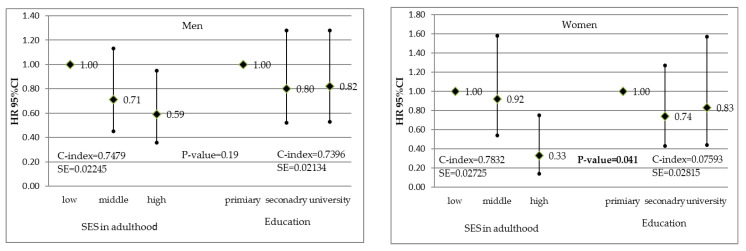
Associations between the socioeconomic status index in adulthood and education, and cardiovascular disease mortality in men and women. Hazard ratios adjusted for age, marital status, obesity, hypertension, hypercholesterolemia, smoking, and diabetes.

The associations between adulthood SES and education alone, and CVD incidence are presented in Figure 1. In men, neither SES index nor education alone were significantly associated with CVD incidence. In women, CVD risk reduction was observed for high SES vs. low SES (adjusted HR = 0.57 and 95% CI = 0.35–0.94) but also for secondary vs. primary education (HR = 0.65 and 95% CI = 0.44–0.96). However, no significant associations were found for university education. The values of the C-index indicated that model discrimination was similar for education and the SES index. Relationships between CVD mortality, and the SES index and education are shown in Figure 2. The lower CVD mortality found in both men and women with high SES compared to low SES was not observed if education alone was considered. Model discrimination was better when the SES index was included instead of education in women.

## 4. Discussion

Childhood SES was not significantly associated with either the risk of CVD or the risk of CVD death at the 10-year follow-up. However, high adulthood SES was confirmed as a protective factor for both fatal and nonfatal CVD. In men, the protective effect of maintaining middle or high SES or upward mobility was observed. In women, the harmful effect of downward mobility might confirm the relationship between low SES and CVD at different levels of exposure. The relationships were independent of the main CVD risk factors. The SES index seemed to be a better measure than education alone as it was significantly related to mortality, which was not observed for education.

Similar to this study, a Finnish-population-based cohort study reported that not poor childhood living conditions but low adult SES was a strong determinant of myocardial infarction incidence and fatality [14]. Similarly, in the GLOBE study in the Netherlands, childhood SES was found to make a modest contribution to the CVD mortality risk, primarily due to its relationship with the classic CVD risk factors [15]. The results of the present study are also consistent with findings from English Longitudinal Study of Ageing (ELSA) in terms of the increased CVD mortality in participants with stable, low SES [16]. In contrast, a study conducted in England did not confirm either the protective effect of upward mobility or the adverse impact of deterioration, which might be explained by the performance of the analysis for sexes combined in the ELSA study. The upward mobility of SES was associated with a lower risk of cardiovascular mortality at the 10-year follow-up in a Korean population [17]. There were some differences in the estimates due to differences in the SES assessment method. The ELSA study included the father’s social class, own education, occupational position, and wealth; the Finnish study included participants’ and parents’ education, occupation, household crowding, home ownership, family type, and income, whereas the Korean study focused on income alone. The latest analysis from Uppsala Birth Cohort Study assessed the SES over four time points and used a latent class analysis to estimate the associations between SES trajectories and mortality, which confirmed that upward mobility reduced the risk of CVD death [18]. However, regardless of the assessment methods, it was not childhood SES but current SES and unfavorable changes in SES that increased the CVD risk, which is consistent with the results of the study indicating that low SES and downward social mobility were associated with poor adult diet quality [19].

In the present study, a cluster method was used to identify SES groups and capture the detailed heterogeneity of the variables describing SES. This approach seems to be suitable for countries in Central Europe, in which social classes are less clearly separated from each other. The cluster method enabled the observation of significant differences in the education level and current professional position between participants with high SES and those with middle or low SES. Sex differences in SES distribution were substantial, and differences in the associations between social mobility and CVD risk between sexes may have been due to baseline differences in SES distribution. Significant associations in men were observed for higher SES categories, in which men were better represented. However, significant associations in women were observed in lower SES, which was more common in women.

However, it seems that even studies that include multiple SES measures cannot examine all potentially important socioeconomic characteristics. The present analysis compared predictive values of the SES index with education alone against CVD incidence and mortality. In terms of the associations with CVD incidence and mortality, the SES index was only slightly better than education alone. However, the improvement in model discrimination was rather small; thus, using education as a simple measure of adulthood SES for the assessment of CVD risk might be justified.

There are several limitations in the interpretation of the presented results that should be considered. First, data on childhood SES were based on the distant recall of amenities available decades earlier, which increases the probability of recall bias and may also be the source of random errors. Also known is the effect of overestimation of SES in childhood, assessed using parental education. Secondly, data on the income of the participants were not available, so only indirect assessment through the declared problems with payments was used. Thirdly, the SES index incorporating several data crucial for SES assessment was used to assure a comprehensive SES assessment; however, the use of this method may be somewhat limited for other authors, as the classification may be, to some extent, dependent on the data. Finally, it should be considered that the register of new CVD cases, based on the participants’ reports in subsequent questionnaires, may have been incomplete.

The study had relevant strengths, including a population-based study sample, prospective design, long follow-up time, and standardized research methods to guarantee the high quality of the data obtained. A comprehensive approach was applied to SES assessment, and data on a higher number of characteristics were included compared with previous studies, for both childhood and adulthood. The comparison of the SEX index with the simplest and most common method of quick SES assessment in terms of CVD prediction was performed, which—to the best of our knowledge—has not been performed before for the population of a region.

## 5. Conclusions

In conclusion, high adulthood SES but not childhood SES may be regarded as a protective factor against CVD in urban populations in high-CVD-risk regions. The effect of the critical period in early life for CVD risk was not observed. In later life, the effect of social mobility was observed on different levels of exposure in both men and women. In men, a protective effect of upward mobility was confirmed, whereas in women, increased CVD risk related to downward mobility was confirmed. CVD prevention may be beneficial if socioeconomic potentials are strengthened in later life.

## Figures and Tables

**Table 1:** Descriptive statistics of the study group by sex.

	Total *N* = 7524	Men *N* = 3591	Women *N* = 3933	*p*-Value
No. of incident CVD, *n* (%)	559 (8.0%)	384 (11.7%)	175 (4.8%)	<0.001
Observation time for CVD incidence, Me (Q1–Q3)	9.16 (8.27–10.77)	8.85 (6.97–10.67)	9.28 (8.48–10.83)	<0.001
No. of CVD deaths, *n* (%)	251 (3.3%)	159 (4.4%)	92 (2.3%)	<0.001
Observation time for CVD mortality, Me (Q1–Q3)	10.08 (9.87–10.75)	10.08 (9.87–10.75)	10.08 (9.87–10.75)	0.04
Age (years), *x* (SD)	56 (6.90)	57 (6.95)	56 (6.84)	<0.001
Marital status (in relationship), *n* (%)	5784 (77.0%)	3136 (87.5%)	2648 (67.5%)	<0.001
Childhood SES, *n* (%)				
Low	2728 (36.9%)	1303 (36.9%)	1425 (37.0%)	0.85
Middle	2728 (36.9%)	1315 (37.2%)	1413 (36.7%)	
High	1930 (26.2%)	914 (25.9%)	1016 (26.4%)	
Adulthood SES, *n* (%)				
Low	2032 (27.0%)	821 (22.9%)	1211 (30.8%)	<0.001
Middle	1813 (24.1%)	849 (23.6%)	964 (24.5%)	
High	3679 (48.9%)	1921 (53.5%)	1758 (44.7%)	
Social mobility, *n* (%)				
Always low	786 (10.8%)	315 (9.1%)	471 (12.4%)	<0.001
Downward mobility	1141 (15.7%)	464 (13.4%)	677 (17.9%)	
Upward mobility	1891 (26.1%)	966 (27.8%)	925 (24.4%)	
Always middle or high	3441 (47.4%)	1730 (49.8%)	1711 (45.2%)	
Smoking, *n* (%)				
Current	2514 (33.6%)	1336 (37.4%)	1178 (30.0%)	<0.001
Former	1983 (26.4%)	1183 (33.0%)	800 (20.4%)	
Never	3005 (40.15%)	1061 (29.6%)	1944 (46.9%)	
Obesity, *n* (%)	1924 (28.9%)	803 (25.3%)	1121 (32.3%)	<0.001
Hypertension, *n* (%)	4029 (58.65%)	2113 (64.5%)	1916 (53.3%)	<0.001
Diabetes, *n* (%)	842 (12.5%)	474 (14.7%)	368 (10.5%)	<0.001
Hypercholesterolemia, *n* (%)	5100 (77.5%)	2424 (77.8%)	2676 (77.3%)	0.63

**Table 2:** Associations between the socioeconomic status index and cardiovascular disease incidence.

	Men				Women			
Childhood SES	HR_a_ (95%CI)	*p*-Value	HR_b_ (95% CI)	*p*-Value	HR_a_ (95% CI)	*p*-Value	HR_b_ (95% CI)	*p*-Value
Low	1.00		1.00		1.00		1.00	
Middle	1.03 (0.82–1.29)	0.817	0.88 (0.68–1.14)	0.335	1.32 (0.94–1.85)	0.108	1.37 (1.00–2.07)	0.060
High	0.77 (0.58–1.02)	0.068	0.74 (0.54–1.02)	0.066	1.06 (0.70–1.61)	0.773	0.91 (0.54–1.51)	0.709
Adulthood SES								
Low	1.00		1.00					
Middle	0.86 (0.66–1.14)	0.296	1.05 (0.77–1.43)	0.777	0.80 (0.57–1.13)	0.205	0.80 (0.53–1.19)	0.261
High	0.68 (0.53–0.89)	0.004	0.86 (0.64–1.15)	0.311	0.40 (0.26–0.62)	<0.001	0.57 (0.35–0.94)	0.026
Social mobility								
Always low	1.00		1.00		1.00		1.00	
Downward mobility	0.85 (0.57–1.25)	0.407	0.85 (0.54–1.34)	0.477	2.02 (1.22–3.33)	0.006	2.24 (1.25–4.02)	0.007
Upward mobility	0.70 (0.50–1.00)	0.048	0.97 (0.65–1.44)	0.866	1.04 (0.62–1.76)	0.879	1.24 (0.67–2.29)	0.489
Always middleor high	0.69 (0.50–0.96)	0.029	0.81 (0.55–1.20)	0.295	0.90 (0.55–1.49)	0.693	1.10 (0.61–1.98)	0.751

a—adjusted for age and marital status. b—adjusted for age, marital status, obesity, hypertension, hypercholesterolemia, smoking, and diabetes.

**Table 3:** Associations between the socioeconomic status index and cardiovascular disease death.

	Men				Women			
Childhood SES	HR_a_ (95% CI)	*p*-Value	HR_b_ (95% CI)	*p*-Value	HR_a_ (95% CI)	*p*-Value	HR_b_ (95% CI)	*p*-Value
Low	1.00		1.00		1.00		1.00	
Middle	1.07 (0.76–1.51)	0.681	0.95 (0.64–1.41)	0.790	1.37 (0.85–2.22)	0.201	1.69 (0.96–2.96)	0.068
High	0.62 (0.39–0.99)	0.045	0.58 (0.34–1.01)	0.055	1.41 (0.82–2.44)	0.215	1.30 (0.65–2.59)	0.463
Adulthood SES								
Low	1.00		1.00		1.00		1.00	
Middle	0.61 (0.41–0.90)	0.014	0.71 (0.45–1.13)	0.153	0.83 (0.53–1.30)	0.420	0.92 (0.54–1.58)	0.771
High	0.44 (0.29–0.65)	<0.001	0.59 (0.36–0.95)	0.030	0.22 (0.11–0.45)	<0.001	0.33 (0.14–0.75)	0.008
Social mobility								
Always low	1.00		1.00		1.00		1.00	
Downward mobility	0.84 (0.51–1.38)	0.486	0.68 (0.37–1.25)	0.218	2.52 (1.24–5.13)	0.011	3.75 (1.43–9.88)	0.007
Upward mobility	0.47 (0.29–0.77)	0.003	0.55 (0.31–0.97)	0.038	1.09 (0.50–2.37)	0.832	1.73 (0.61–4.90)	0.302
Always middle or high	0.46 (0.30–0.73)	0.001	0.50 (0.29–0.84)	0.010	0.96 (0.46–2.02)	0.923	1.61 (0.59–4.36)	0.348

a—adjusted for age and marital status. b—adjusted for age, marital status, obesity, hypertension, hypercholesterolemia, smoking, and diabetes.

## Data Availability

The datasets used and/or analyzed in the current study may be available from the corresponding author upon reasonable request.

## Supplementary Materials

The following supporting information can be downloaded at: https://www.mdpi.com/article/10.3390/ijerph192215411/s1, Table S1. SES clusters and distribution of characteristics used for SES index. Table S2. Distributions of age, marital status, smoking, obesity, hypertension, diabetes, hypercholesterolemia, by SES in men. Table S3. Distributions of age, marital status, smoking, obesity, hypertension, diabetes, hypercholesterolemia, by SES in women.
