# Supplementary material for: Changes in Socioeconomic Status as Predictors of Cardiovascular Disease Incidence and Mortality: A 10-Year Follow-Up of a Polish-Population-Based HAPIEE Cohort
Source: Int J Environ Res Public Health. 2022 Nov 21;19(22):15411. doi: 10.3390/ijerph192215411 (PMC9693797; doi:10.3390/ijerph192215411)
Supplement: Supplementary file 1 [file ijerph-19-15411-s001.zip › ijerph-2008537-supplementary.pdf]

Table S1. SES clusters and distribution of characteristics used for SES index.

	**Childhood SES**			
Variable		Low	Middle	High
The highest completed level of education of parents	Father			
	lower than secondary school	2728 (100%)	2203 (80.7%)	192 (10.0%)
	secondary school	0	439 (16.1%)	916 (47.5%)
	university degree	0	86 (3.2%)	822 (42.5%)
	Mather			
	lower than secondary school	2728 (100%)	2668 (97.8%)	0
	secondary school	0	56 (2.1%)	1567 (81.2%)
	university degree	0	3 (0.1%)	353 (18.8%)
Index measuring amenities in childhood, calculated based on questions about which of the following items participants had at age 10 (cold tap water, hot tap water, radio, fridge, own kitchen and own toilet). Median (Q1-Q), range:0-6		2 (2-4)	4 (2-5)	5 (4-6)
	**Adulthood SES**			
		Low	Middle	High
Highest completed level of education	lower than secondary school	971 (47.8%)	624 (34.4%)	680 (18.5%)
	secondary school	846 (41.6%)	774 (42.7%)	1305 (35.5%)
	university degree	215 (10.6%)	415 (22.9%)	1694 (46.1%)
Current professional position	owner of a company	0	0	555 (15.1%)
	employed,	303 (14.9%)	0	2884 (78.4%)
	retired or unemployed	1729 (85.1%)	1813 (100%)	240 (6.5%)
Index of household amenities based on the number of valuable items which a participant had in his household (microwave, washing machine, cottage house etc.) . Median (Q1-Q3), range 0-12.		5 (4-7)	6 (5-8)	8 (6-9)
Current financial situation (based on two questions:				
"How often does it happen not to have enough money for food which you and your family need?"	all the time	116 (5.7%)	0	0
	often	539 (26.5%)	0	58 (1.6%)
	sometimes	687 (33.8%)	0	230 (6.3%)
	rarely	466 (22.9%)	0	394 (10.7%)
	never	224 (11.0%)	1813 (100%)	2997 (81.5%)
Do you have any difficulties with paying bills	all the time	155 (7.6%)	0	0
	often	460 (22.6%)	0	129 (3.5%)
	sometimes	621 (30.6%)	0	376 (10.2%)
	rarely	376 (18.5%)	0	391 (10.6%)
	never	420 (20.7%)	1813 (100%)	2783 (75.7%)

Table S2. Distributions of age, marital status, smoking, obesity, hypertension, diabetes, hypercholesterolemia, by SES in men.													
Men	Childhood SES				Adulthood SES				Social mobility				
	Low	Middle	High	P value	Low	Middle	High	P value	Always low	Downward mobility	Upward mobility	Always middle or high	P value
Age [years], x(SD)	58 (6.79)	57 (7.10)	55 (6.63)	<0.001	58 (6.98)	63 (5.26)	54 (5.75)	<0.001	59 (6.94)	57 (6.91)	58 (6.69)	56 (6.70)	<0.001
Marital status (in relationship), n(%)	1149 (88.3)	1148 (87.6)	784 (85.9)	0.24	645 (78.8)	753 (88.7)	1738 (90.7)	<0.001	247 (78.4)	366 (79.2)	886 (91.8)	1536 (88.9)	<0.001
Smoking, n(%)													
Current	443 (34.1)	513 (39.1)	357 (39.1)	0.067	408 (50.0)	234 (27.7)	694 (36.2)	<0.001	151 (48.2)	238 (51.6)	285 (29.6)	620 (35.9)	<0.001
Former	449 (34.6)	418 (31.9)	291 (31.9)		234 (28.7)	328 (38.8)	621 (32.4)		93 (29.7)	128 (27.8)	349 (36.2)	568 (32.9)	
Never	406 (31.3)	380 (29.0)	264 (29.0)		174 (21.3)	284 (33.6)	603 (31.4)		69 (22.0)	95 (20.6)	329 (34.2)	539 (31.2)	
Obesity, n(%)	316 (27.2)	297 (25.5)	167 (20.8)	0.005	180 (25.2)	215 (27.8)	408 (24.1)	0.16	71 (26.2)	97 (24.0)	241 (27.7)	356 (23.3)	0.11
Hypertension, n(%)	813 (67.9)	770 (64.0)	502 (60.4)	0.002	485 (66.7)	586 (72.9)	1042 (59.6)	<0.001	195 (71.4)	267 (63.7)	601 (66.6)	980 (61.9)	0.007
Diabetes, n(%)	184 (15.7)	186 (15.7)	101 (12.3)	0.067	132 (18.1)	156 (19.7)	186 (10.9)		59 (21.2)	68 (16.4)	120 (13.7)	215 (13.8)	0.007
Hypercholesterolemia, n(%)	872 (76.6)	897 (78.3)	616 (78.7)	0.47	525 (74.8)	590 (77.6)	1309 (79.1)	0.07	188 (70.9)	305 (76.3)	670 (78.3)	1181 (78.9)	0.04

Table S3. Distributions of age, marital status, smoking, obesity, hypertension, diabetes, hypercholesterolemia, by SES in women.													
Women	Childhood SES				Adulthood SES				Social mobility				
	Low	Middle	High	P value	Low	Middle	High	P value	Always low	Downward mobility	Upward mobility	Always middle or high	P value
Age [years], x(SD)	57 (6.81)	56 (6.84)	55 (6.61)	<0.001	58 (6.92)	62 (5.35)	53 (5.08)	<0.001	58 (6.87)	57 (6.88)	57 (6.75)	55 (6.63)	<0.001
Marital status (in relationship), n(%)	985 (69.2)	954 (67.6)	664 (65.7)	0.19	708 (58.6)	672 (69.8)	1268 (72.3)	<0.001	285 (60.5)	390 (57.8)	680 (73.6)	1201 (70.4)	<0.001
Smoking, n(%)													
Current	383 (27.0)	444 (31.6)	333 (32.8)	<0.001	411 (34.1)	194 (20.2)	573 (32.7)	<0.001	145 (30.9)	249 (36.9)	227 (24.6)	513 (30.1)	<0.001
Former	262 (18.4)	290 (20.6)	232 (22.9)		220 (18.2)	197 (20.5)	383 (21.8)		77 (16.4)	133 (19.7)	182 (19.7)	379 (22.2)	
Never	776 (54.6)	673 (47.8)	449 (44.3)		575 (47.7)	571 (59.4)	798 (45.5)		247 (52.7)	292 (43.3)	514 (55.7)	815 (47.7)	
Obesity, n(%)	465 (36.3)	413 (33.0)	213 (24.2)	<0.001	426 (40.3)	320 (36.6)	375 (24.2)	<0.001	186 (45.1)	216 (36.5)	270 (32.1)	399 (26.5)	<0.001
Hypertension, n(%)	752 (56.7)	706 (54.5)	423 (46.6)	<0.001	672 (60.4)	579 (64.2)	665 (42.0)	<0.001	282 (64.8)	357 (57.5)	454 (52.6)	749 (48.5)	<0.001
Diabetes, n(%)	146 (11.3)	133 (10.6)	75 (8.5)	0.09	146 (13.7)	134 (15.1)	88 (5.7)	<0.001	55 (13.3)	78 (13.1)	85 (10.0)	127 (8.4)	0.001
Hypercholesterolemia, n(%)	1017 (79.6)	951 (76.2)	659 (75.2)	0.03	823 (78.6)	719 (82.6)	1134 (73.4)	<0.001	336 (82.0)	446 (76.4)	657 (78.3)	1134 (75.3)	0.03
